# Genome-Wide Characterization of Genetic Variation in the Unicellular, Green Alga *Chlamydomonas reinhardtii*

**DOI:** 10.1371/journal.pone.0041307

**Published:** 2012-07-25

**Authors:** Hyosik Jang, Ian M. Ehrenreich

**Affiliations:** Molecular and Computational Biology Section, University of Southern California, Los Angeles, California, United States of America; North Carolina State University, United States of America

## Abstract

*Chlamydomonas reinhardtii* is a model system for studying cilia, photosynthesis, and other core features of eukaryotes, and is also an emerging source of biofuels. Despite its importance to basic and applied biological research, the level and pattern of genetic variation in this haploid green alga has yet to be characterized on a genome-wide scale. To improve understanding of *C. reinhardtii*'s genetic variability, we generated low coverage whole genome resequencing data for nearly all of the available isolates of this species, which were sampled from a number of sites in North America over the past ∼70 years. Based on the analysis of more than 62,000 single nucleotide polymorphisms, we identified two groups of isolates that represent geographical subpopulations of the species. We also found that measurements of genetic diversity were highly variable throughout the genome, in part due to technical factors. We studied the level and pattern of linkage disequilibrium (LD), and observed one chromosome that exhibits elevated LD. Furthermore, we detected widespread evidence of recombination across the genome, which implies that outcrossing occurs in natural populations of this species. In summary, our study provides multiple insights into the sequence diversity of *C. reinhardtii* that will be useful to future studies of natural genetic variation in this organism.

## Introduction

*Chlamydomonas reinhardtii* is a haploid, unicellular eukaryote that is an important model system in biology (as reviewed in [1] and elsewhere). *C. reinhardtii* is well-known for having both animal-like and plant-like organelles – cilia and chloroplasts, respectively – that probably originated from a common animal-plant ancestor in the past [2]. Due to the unique characteristics of *C. reinhardtii*, a large fraction of the research in this organism has focused on understanding photosynthesis, as well as the structure and function of flagella [3], [4]. However, *C. reinhardtii* has also been studied in a number of other contexts, including in research focused on biofuels [5], evolutionary ecology (e.g., [6]), and experimental evolution (e.g., [7]).

Although many *C. reinhardtii* strains are available from stock centers, most of these strains originate from a single isolate of the species, 137c, that G.M. Smith isolated near Amherst, Massachusetts in 1945 [1], [8]. More recently, the *Chlamydomonas* research community has sampled additional strains from nature [9], [10], [11], [12], primarily to facilitate the use of crosses in localizing and identifying mutations in the 137c strain background [13], [14]. By aiding in the genetic mapping of induced and spontaneous mutations of biological interest (e.g., [15]), natural genetic variation has played a critical role in molecular genetics research in *C. reinhardtii*.

However, the full potential of natural genetic variation to provide insights into the biology of *C. reinhardtii* has yet to be exploited. As shown in other model systems, natural genetic variation can serve as a valuable resource for identifying novel genes and gene functions (e.g., [16], [17]), understanding the genetic basis of complex phenotypes (e.g., [18], [19], [20], [21], [22]), and determining the molecular mechanisms of adaptation (e.g., 23,24). With respect to *C. reinhardtii*, natural genetic variants that segregate among isolates of this species may affect phenotypes that interest scientists working with this organism. By characterizing these variants and the genes in which they reside, novel insights may be gained into the molecular biology and evolution of *C. reinhardtii*.

*C. reinhardtii* possesses many characteristics that could make it a useful model organism for population genomics and quantitative genetics. Unlike most commonly studied eukaryotes, *C. reinhardtii* is haploid, suggesting the efficacy of natural selection in this organism may be high [25]. *C. reinhardtii* possesses two mating types – *mt-* and *mt+* – that are determined by a specific mating-type locus [1]. This organism can grow asexually or sexually, facilitating both the maintenance of immortalized strain lineages and the crossing of different isolates [1]. *C. reinhardtii* has a ∼120 Megabase [Mb] nuclear genome that is distributed across 17 chromosomes [2]. In addition, *C. reinhardtii* possesses a favorable average recombination rate of ∼100 kilobases [kb]/centiMorgan [cM] [2].

Presently, about a dozen wild isolates of *C. reinhardtii* are publicly available. Previous genotyping experiments involving restriction fragment length polymorphisms [9], [10], [12], a small number of resequenced nuclear loci [8], [26], [27], or extensive chloroplast resequencing [28] established that these different isolates of *C. reinhardtii* are not isogenic, and suggested that the species carries ∼2.7 single nucleotide polymorphisms (SNPs) every 100 bases in the nuclear genome [14]. Although these prior studies provided valuable information regarding the sequence diversity of *C. reinhardtii*, the level and pattern of genetic diversity in this species has yet to be comprehensively assessed.

**Figure 1:**
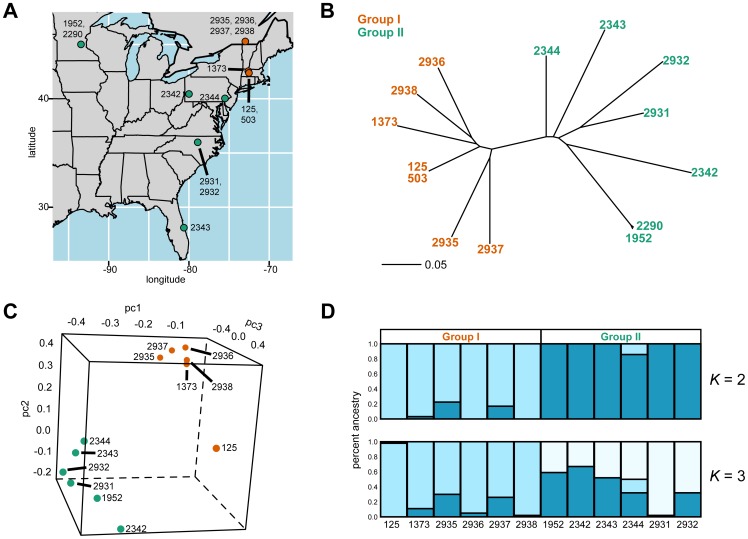
Sampling locations, genealogy, and population structure of *C. reinhardtii* isolates. A map depicting where each of the sequenced strains was isolated is shown in **A** [8], [10], [11], [12], [46]. Each of the unique sampling locations is plotted on the map. We color the isolates according to the groups described in the main text. Group I isolates are in orange, and Group II isolates are in green. The isolates that were obtained from a location are written next to the circle for that location. 137c, the progenitor to CC-125 and CC-503, was obtained in 1945. CC-1373 was also isolated in 1945. The rest of the strains were collected in the 1980s and 1990s. A neighbor-joining tree of the strains is shown in **B**, with the genetic distances determined as the fraction of examined SNPs that were different between two strains. The loadings of the strains on the first three principal components is shown in **C**. **D** displays STRUCTURE results from *K = 2* and *K = 3*. The barcharts indicate how much of the isolates' genomes are derived from each of the *K* ancestral subpopulations in the model.

To provide new insights into the natural genetic variation of *C. reinhardtii*, we conducted a genome-wide analysis of its genetic diversity. We resequenced at low genomic coverage most of the available *C. reinhardtii* isolates, which originate from multiple states in the US (Florida, Massachusetts, Minnesota, North Carolina, and Pennsylvania), as well as a single field in Quebec (**Fig. 1A**; [1], [8], [9], [10], [11], [12]). We identified a large number of genetic variants throughout the genome, and used these polymorphisms to study multiple previously unknown aspects of *C. reinhardtii*'s sequence variation. In particular, we determined the genetic relationships and population structure of the isolates, identified potential signatures of natural selection on the genome, and found evidence for outcrossing during the history of the species. Our study provides insights into the population genetics of *C. reinhardtii* that will aid future studies of natural genetic variation in this model organism.

## Results

### Low coverage genome sequencing of most *C. reinhardtii* isolates

We obtained nearly all of the available natural isolates of this species (12 total; **Fig. 1A**). In addition, we acquired CC-125 and CC-503, which are both descended from the *C. reinhardtii* reference isolate 137c (**Fig. 1A**). CC-125 is regarded as the wild type 137c genotype and is employed in many mutational screens in this species [29], while CC-503 was used to produce the *C. reinhardtii* reference genome [2]. We generated Illumina sequencing data at an average of ∼10-fold genomic coverage per strain. Among the strains, we identified 62,474 SNPs, 15,281 indels, and 10,191,386 invariant sites that were sequenced in every individual, leading to a marker discovery rate of ∼6 SNPs per kb (**Methods**).

### Population structure of the isolates

To better understand the demography of *C. reinhardtii*, we examined the SNP data for evidence of population structure. We made a neighbor-joining tree of the isolates, and identified two clades (**Fig. 1B**; **Methods**). CC-125, CC-503, CC-1373, CC-2935, CC-2936, CC2937, and CC-2938 formed the first clade (‘Group I’), while CC-1952, CC-2290, CC-2342, CC-2343, CC-2344, CC-2931, and CC-2932 formed the second clade (‘Group II’). We found that CC-125 and CC-503 were nearly identical, as was expected given their common ancestry (**Fig. 1B**). In addition, we found that two strains obtained from the same field in Minnesota [29] – CC-1952 and CC-2290 – were very similar at the sequence level (**Fig. 1B**). To prevent any artifacts that could arise from examining redundant genotypes, we excluded CC-503 and CC-2290 from subsequent analyses.

We used additional methods to study the population structure among the isolates. Results from both Principal Components Analysis (PCA) and the program STRUCTURE [30] were consistent with the neighbor-joining tree (**Figs. 1C and 1D**; **Methods**). PCA is a multivariate statistical approach that is often used to identify signatures of population structure in large-scale SNP genotyping datasets (as described in [31] and elsewhere). With PCA, the groups could be clearly distinguished when we analyzed the loadings of the isolates for the first two or three principal components (**Fig. 1C**). The PCA results also suggested that CC-125, the reference strain of this species, is somewhat different at the genetic level from the other Group I isolates, possibly reflecting its long-term cultivation in the lab (**Fig. 1C**). Furthermore, principal component 3 was correlated with the latitudes at which the isolates were collected, with the signature most pronounced in Group II (Group II only: Spearman's ρ = 0.83, p = 0.021; all isolates: Spearman's ρ = 0.69, p = 0.011). This indicates that a number of genetic variants in this species may be distributed across a north-south cline (**Fig. 1** and **Fig. S1**).

The STRUCTURE algorithm estimates the relative contribution of each of *K* putative ancestral subpopulations to the genomes of individuals in a sample [30]. In the STRUCTURE analysis, the most likely model included two ancestral subpopulations (*K* = 2), and implementations of the program with *K* = 2 or *K* = 3 gave ancestry assignments for the strains that were consistent with the neighbor-joining tree and PCA (**Fig. 1D**). Thus, two groups of isolates were present among the sequenced strains.

We examined the level of genetic differentiation between the two groups, and found that only ∼1.3% of the identified SNPs showed fixed differences between the subpopulations (F_ST_ = 1). This suggests that the two populations may share a recent evolutionary origin. We also note that the geographic bounds of the *C. reinhardtii* populations may be temporally dynamic, as CC-125 and CC-1373, which were sampled from Massachusetts in 1945, were more closely related to recent isolates from Quebec than to recent isolates from the US (**Fig. 1**).

### Levels of genetic variation differ across site classes

**Figure 2:**
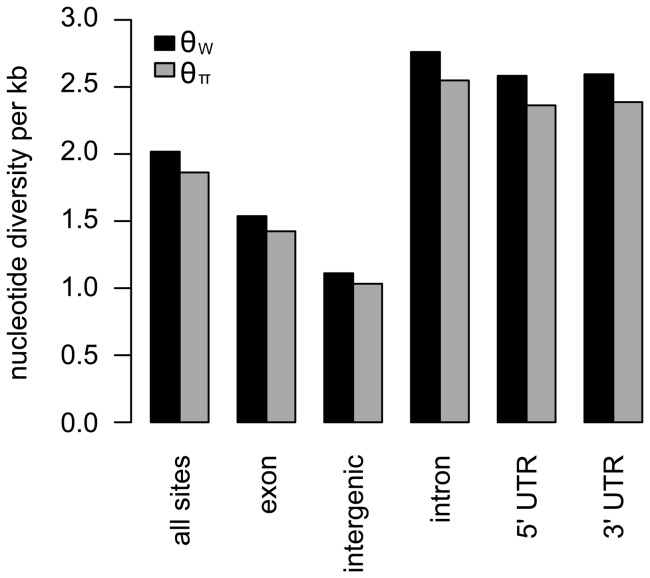
Distributions of genetic variants and nucleotide diversity across site classes. Two estimates of the population mutation rate – θ_π_ and θ_W_ – are shown by site class.

We determined the levels of genetic variation at different site classes in the *C. reinhardtii* genome. SNPs were detected across a large number of annotation classes, with 25.9% in exons, 9.5% in intergenic regions, 33.4% in introns, 11.4% in 5′ untranslated regions (UTRs), and 19.8% in 3′ UTRs. We used the identified SNPs to estimate *C. reinhardtii*'s nucleotide diversity (i.e., the population mutation rate θ, which is equal to 2N_e_μ, where N_e_ equals the effective population size and μ equals the neutral mutation rate; **Methods**). We found that measures of sequence variation based on segregating sites (θ_W_) [32] and average number of pairwise sequence differences (θ_π_) [33], [34] were 0.0021 per base and 0.0019 per base, respectively. Across site classes, estimates of θ_π_ were generally ∼8% lower than estimates of θ_W_ (**Fig. 2**), indicating a slight skew towards low frequency alleles throughout the genome. We observed a wide range of nucleotide diversities across site classes, with the lowest value of θ_W_ observed in intergenic regions (0.0011) and the highest value of θ_W_ observed in introns (0.0028). Exons also showed reduced sequence variation relative to introns and UTRs (θ_W_ = 0.0015 for exons vs. θ_W_ = 0.0028 for introns and θ_W_ = 0.0026 for UTRs), although nucleotide diversity was ∼38% higher in exons than in intergenic regions.

### Heterogeneity in nucleotide diversity across the genome

**Figure 3:**
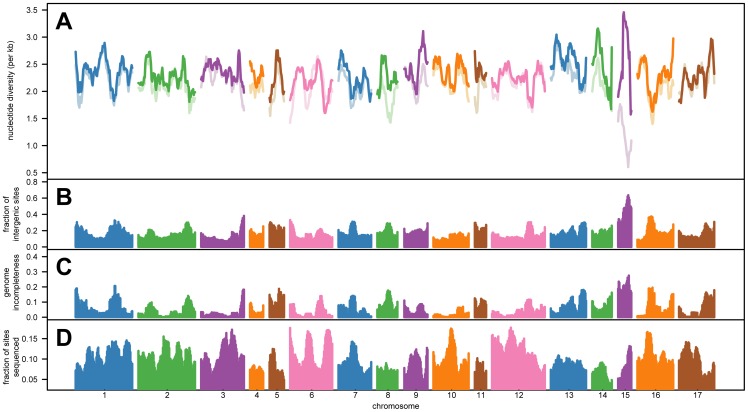
Analyses of levels of nucleotide diversity across the genome. In **A**, we plotted the observed levels of sequence diversity in 100 kb windows throughout the genome, with color-coding by chromosome. The 10-window local averages for θ_W_ are shown in colored lines for each of the chromosomes. The lighter lines show results with intergenic DNA included, while the darker lines show results with intergenic DNA excluded. In **B**, the fraction of intergenic DNA sites in a genomic window is plotted. In **C**, the fraction of sites in the reference genome sequence that are coded as ambiguous bases within a window is shown. In **D**, the fraction of sites within a window at which every strain was sequenced is depicted. Vertical bars that are color-coded by chromosome are used to show the measurements in **B**, **C**, and **D**.

We determined the extent to which levels of genetic diversity vary within and between chromosomes in the *C. reinhardtii* genome. To do this, we split the genome into equally sized and non-overlapping windows of 100 kb, and examined local levels of nucleotide diversity across the genome, as measured by θ_W_ (we note that other window sizes produced similar results). We first analyzed levels of nucleotide diversity across the genome in the two subpopulations described above. We found that local levels of genetic variation in the genome tended to be higher among the Group II strains, suggesting that this subpopulation has a larger effective population size than the Group I subpopulation (**Fig. S2**). Because of our small sample size, we combined the data from the two subpopulations to increase our statistical power to detect unusual patterns of nucleotide diversity throughout the genome. When we did this, we observed substantial heterogeneity in levels of observed genetic variation (**Fig. 3A**), with nucleotide diversity showing a range between ∼0 and 0.0048 per base. 16 of the chromosomes exhibited average levels of nucleotide diversity that were within 13% of the genome-wide value of θ_W_. However, Chromosome 15 showed a highly significant, ∼41% reduction in sequence variation relative to the rest of the genome (ANOVA: F = 45.668, d.f.  = 1, p = 2.34×10^−11^; **Fig. 3A**).

We tried to identify factors causing the reduction of nucleotide diversity on Chromosome 15, and found that this chromosome is unusual relative to the other chromosomes. As described above, intergenic sites exhibit reduced genetic diversity in comparison to other functional categories of sites (**Fig. 2**). Based on the present genome annotation, nearly half (48%) of Chromosome 15 is comprised of intergenic sites (**Fig. 3B**). In contrast, all of the other chromosomes consist of between 12% and 28% intergenic sites (**Fig. 3B**). To better understand the effect of intergenic regions on the observed genome-wide heterogeneity in nucleotide diversity, we re-examined the data by excluding intergenic sites from measurements of local levels of genetic variation. When we did this, Chromosome 15 showed levels of nucleotide diversity that were similar to or greater than the rest of the genome (**Fig. 3A**), indicating that the observation of reduced diversity on this chromosome was associated with its large amount of intergenic DNA.

The effect of intergenic DNA on measurements of genetic diversity for Chromosome 15 may be a technical artifact. We assessed the completeness of the reference genome within each genomic window (as defined as the fraction of ambiguous bases in the reference sequence within a 100 kb chromosomal segment), and found substantial heterogeneity in the quality of the reference sequence (**Fig. 3C**). Regions containing large amounts of intergenic DNA often also showed high levels of incompleteness, with Chromosome 15 being the most extreme (**Fig. 3C**). Short sequencing reads are less likely to properly align to regions of the genome that are incomplete, and this problem will be exacerbated when genetic variants are present. Consistent with this explanation, disproportionately fewer intergenic SNPs were detected on Chromosome 15 than the other chromosomes, even though Chromosome 15 did not appear different from the rest of the genome at other categories of functional sites (**Table S1**).

Even though technical factors likely play a role in our measurements of genetic diversity on Chromosome 15, we cannot rule out an additional contribution by biological factors, such as negative selection on intergenic regions. For instance, the amount of data generated for Chromosome 15 was comparable to the other chromosomes (**Fig. 3D**), suggesting that our ability to detect SNPs on Chromosome 15 could be similar to the rest of the genome. We also note that intergenic sites had a genome-wide effect on measurements of nucleotide diversity. When all chromosomes were considered, the fraction of intergenic sites in a window showed a highly significant negative correlation with nucleotide diversity (Spearman's ρ = −0.29, p<2.2×10^−16^), and explained ∼9.8% of the variance in nucleotide diversity in a simple linear regression. Thus, how intergenic DNA is distributed throughout the *C. reinhardtii* genome influences our observations of genetic diversity, although additional data are necessary to fully determine the contributions of biological and technical factors to this signature.

### Analysis of the minor allele frequency spectrum

**Figure 4:**
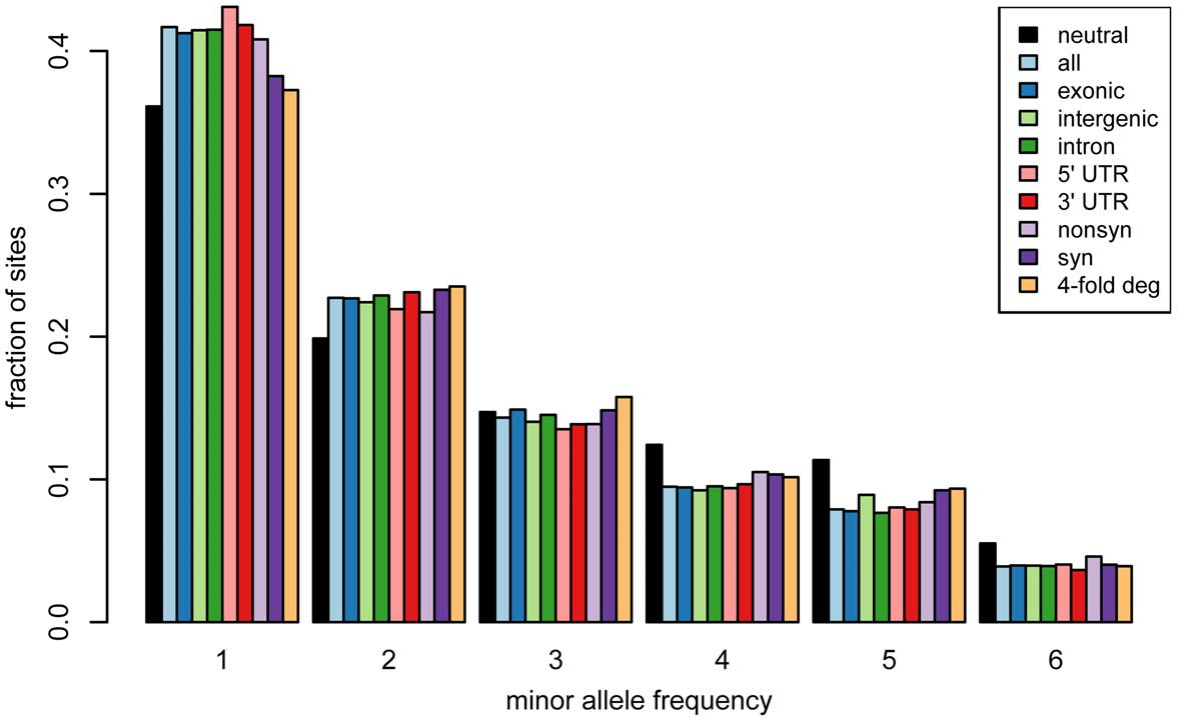
Minor allele frequency spectrum. A legend is provided that shows how the colored bars correspond to different site classes.

We studied the minor allele frequency spectrum of the identified SNPs to obtain additional insights into the genetic diversity of *C. reinhardtii*. The allele frequency spectrum can provide information about the types of historical events that have influenced genetic diversity in a species, as well as the extent of natural selection on different site classes (e.g., [35]). We measured the species-wide spectrum for all of the SNPs, and compared it to the expectation for a neutrally evolving population that has not experienced selection or demographic events (**Fig. 4**). Relative to the theoretical spectrum for 12 individuals [34], [35], [36], we observed an excess of sites with low minor allele frequencies. This can be a signature of recent population expansion, which causes an increase in rare variants relative to the expectation for a population that is at equilibrium. We then examined the spectra of SNPs located in intergenic regions, introns, 5′ UTRs, 3′ UTRs, and exons. We further split the exonic SNPs into groups of nonsynonymous and synonymous variants, and also looked at four-fold degenerate positions in exons. Relative to putatively neutral sites (i.e., synonymous and four-fold degenerate sites), we observed that all of the other site classes had spectra that were skewed towards low frequencies, indicating negative selection against deleterious polymorphisms in these site classes (**Fig. 4**). This result is consistent with findings in other species, including mammals [37] and flies [38], that suggest functional site classes outside of coding regions often experience strong selective constraint.

### Pattern of linkage disequilibrium

**Figure 5:**
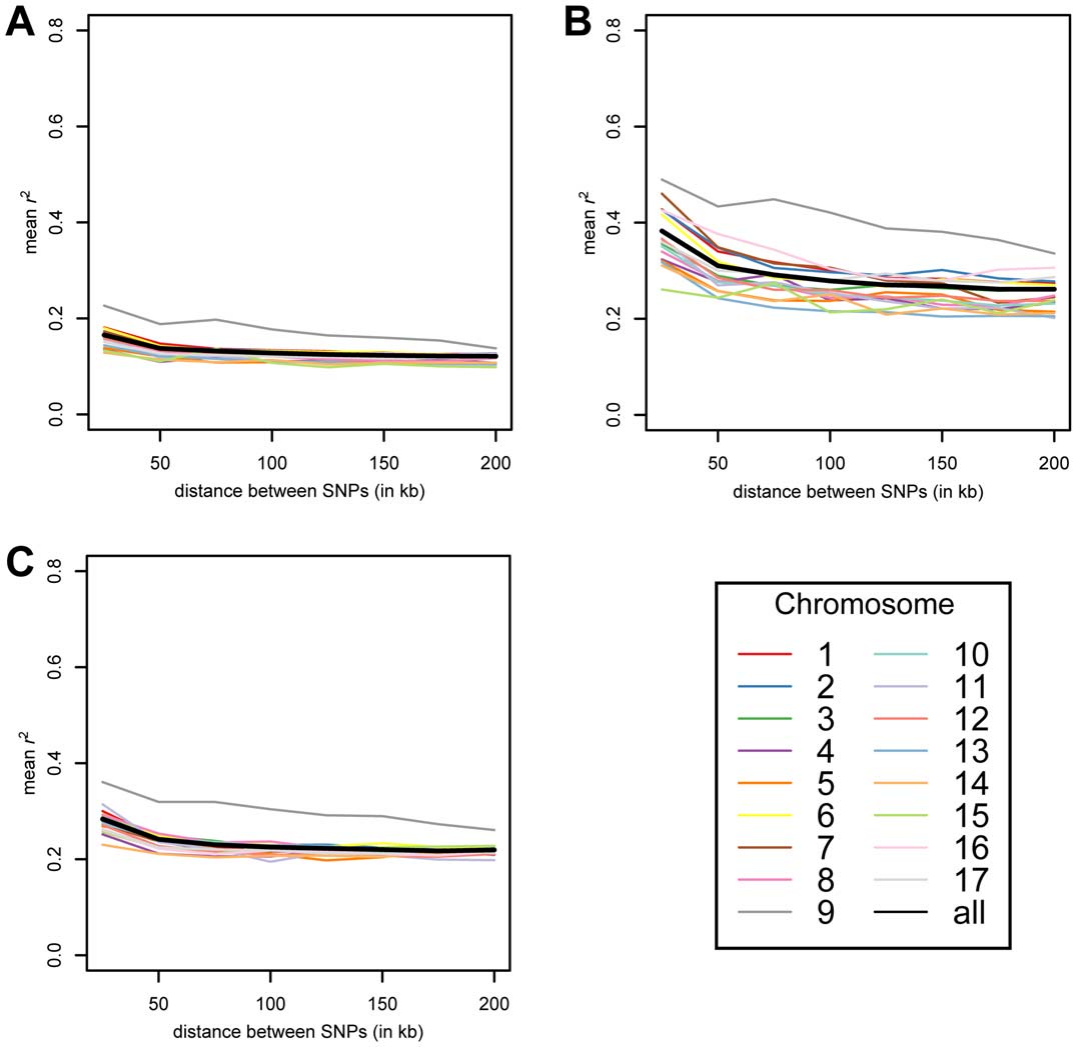
Decay of linkage disequilibrium. Average values of *r* ^2^ within non-overlapping 25 kb windows are plotted. Values for the entire isolate panel are shown in **A**, while the Groups I and II subpopulations are shown in **B** and **C**, respectively. The genome-wide values are depicted in black, while the chromosomes are shown in color. The color key for the chromosomes is shown in the lower right corner.

Linkage disequilibrium (LD) is the nonrandom association of alleles at different sites in the genome. We sought to examine the rate at which LD decays at linked sites in the sample using the *r* ^2^ metric [39]. At first, we measured the extent of LD among the full collection of isolates. Although a number of linked and unlinked SNPs in perfect LD were observed, we found that *r* ^2^ decayed to ∼0.17 within 25 kb when the values for all pairs of linked sites were averaged (**Fig. 5A**). We observed similar rates of decay of LD from maximum to baseline levels when we examined only Group I (**Fig. 5B**) or Group II isolates (**Fig. 5C**). However, the subpopulations exhibited different baseline levels of LD relative to the entire sample and each other: the Group I strains showed a baseline *r* ^2^ between distantly linked sites of ∼0.3, while baseline *r* ^2^ between distantly linked sites was ∼0.24 among the Group II strains.

We also looked at the rate of decay of LD at the level of individual chromosomes. Most chromosomes exhibited rates of decay of LD that were similar to genome-wide values (**Fig. 5**). However, Chromosome 9 displayed unusually high LD when all of the isolates or either of the groups of isolates were considered (**Fig. 5**). A couple of domains of elevated haplotype structure were visible among the isolates (**Fig. S3**), suggesting a common cause for high LD on Chromosome 9 in the two subpopulations. The regions with high LD did not appear to be due to reduced local recombination. Chromosome 9, including the region with high LD, showed an above average level of recombination relative to the rest of the genome in a controlled cross of two *C. reinhardtii* strains (**Fig. S4**; **Methods**). The occurrence of partial selective sweeps is another possible explanation for the presence of long and incompletely fixed haplotypes in these regions. Because the regions of high LD are large and span more than a megabase, it is difficult to speculate which gene or genes in these regions might have been the target of any selection.

### Evidence for outcrossing in natural populations of *C. reinhardtii*

Recombination is the primary factor that causes LD to break down. In a haploid species like *C. reinhardtii*, recombination occurs when two individuals of opposite mating types encounter each other, cross, and produce offspring. For any recombination event to be detectable in cross progeny, two mates must carry different alleles of markers that flank a recombination event. We examined the data for pairs of linked sites that exhibited evidence of recombination either at the whole species level or within the subpopulations. We found that a large number of SNP pairs distributed on each of the chromosomes showed evidence of recombination based on the segregation of all four possible combinations of two linked SNPs [40]. Specifically, we identified ∼23.5 million pairs of sites when all isolates were considered, ∼4.6 million pairs of sites when only the Group I isolates were considered, and ∼7.7 million pairs of sites when only the Group II isolates were considered. These results, as well as the findings from our analysis of LD, provide evidence that *C. reinhardtii* outcrosses in nature.

We attempted to estimate the amount of outcrossing that occurred during the evolutionary history of the isolates. To do this, we followed the example of Ruderfer et al. [41]. In brief, when the recombination rate per generation (*r*) is known for a species and an estimate of the population recombination rate (ρ = 2N_e_ *r*) has been obtained for a population genetics sample from that species, one can estimate how many generations of outcrossing have contributed to the sample (**Methods**). Using the program LDHat [42], we estimated ρ for each chromosome using two approaches – based on the distribution of pairwise differences across the isolates and by composite likelihood analysis. We estimated the genome-wide average value of ρ to be 218 using the former approach, while the latter approach consistently gave estimates that were nearly an order of magnitude higher. We used the more conservative estimate of ρ in our analysis. Assuming a previously published recombination rate of ∼0.01 cM/kb [2], [13], [14], our results indicate that ∼1,089 generations of outcrossing occurred during the history of the examined isolates. Although additional information about the effective population size of *C. reinhardtii* is needed to accurately determine the rate of outcrossing in this species, our results suggest that a large number of mating events have taken place in natural populations of this organism.

## Discussion

### Genome-wide identification of genetic variants leads to new insights regarding natural variation in *C. reinhardtii*

*C. reinhardtii* is a widely studied model system in biology, but the genetic diversity of this species is not well understood. To characterize the sequence variation of *C. reinhardtii*, we generated genome-wide resequencing data for most of the available isolates of this species. We found that nucleotide diversity in *C. reinhardtii* is ∼0.0021 per base. This value is slightly lower than in other unicellular, eukaryotic model systems, such as *Saccharomyces cerevisiae* and *Schizosaccharomyces pombe*, which possess nucleotide diversities of ∼0.0057 [43], [44] and ∼0.007 [45] per base, respectively, but suggests that *C. reinhardtii* has the raw material to serve as a system for studying natural genetic variation. In an attempt to address multiple basic questions about the population genetics of *C. reinhardtii*, we used the identified genetic variants to determine the level and pattern of sequence diversity across the examined isolates and their genomes. In the following subsections, we elaborate upon a number of previously unknown features of genetic variation in *C. reinhardtii*.

### *C. reinhardtii* exhibits population subdivision, and shows genetic diversity on a local geographic scale

We found that the sequenced isolates represent two subpopulations of *C. reinhardtii*, which we refer to as Groups I and II (**Fig. 1**). Group I consisted of the reference strain and another isolate – CC-1373 – that were obtained from Massachusetts in 1945, as well as four strains that were isolated from a field in Quebec in the early 1990s [1], [8]. Despite being interfertile with other isolates, CC-1373 had originally been described as a distinct species of *Chlamydomonas* due to its unusual morphology [46]. Sequencing of a small number of loci has since shown that CC-1373 is similar to other *C. reinhardtii* isolates at the genetic level, and has led CC-1373 to be regarded as a representative of *C. reinhardtii* [1], [8]. Our results indicate that CC-1373 is certainly a member of *C. reinhardtii*.

Group II was also comprised of strains from the US, although these isolates were collected decades after 137c and CC-1373 from sites that span a very broad geographic range [9], [10], [11]. Given that some of the Group II isolates were sampled from locations that are close to where CC-125 and CC-1373 were collected (**Fig. 1A**), it is possible that the geographic bounds of the Group I and II subpopulations have changed since the initial isolation of *C. reinhardtii*. We also identified suggestive evidence of substructure among Group II isolates – genetic variation among these strains appears to exhibit a relationship with latitude. This may reflect neutral demographic effects, such as isolation-by-distance, or adaptation to environmental factors, but understanding this pattern in more detail will require the collection and genotyping of more isolates.

Our results also show that local populations of *C. reinhardtii* can be genetically variable. One piece of evidence supporting this comes from four Group I isolates that were obtained from a single field in Quebec, Canada [12] (**Fig. 1A**). These strains are not clonal (**Fig. 1**), but rather show a level of nucleotide diversity (θ_W_) of 0.0014 per base, which is ∼66% of the species-wide value. The Quebec isolates are not the only local population that exhibits genetic diversity in the data: CC-2931 and CC-2932, which were collected from the same field in North Carolina and are both in Group II, also possess a number of sequence differences. Considering just CC-2931 and CC-2932, we obtained an estimate of θ_W_ that was equal to 0.0013 (∼62% of the species-wide value). These results suggest that local populations of *C. reinhardtii* may provide valuable study systems for geneticists and evolutionary biologists.

### Measurements of LD and recombination provide insights into positive selection and outcrossing

Linkage disequilibrium is a topic of interest in population genomics studies for multiple reasons: it can provide valuable insights into the history of a population, and is a central consideration for genotype-phenotype association mapping. We showed that LD decays rapidly throughout the genome and exhibits moderately low baseline levels in *C. reinhardtii*, particularly when all of the sequenced isolates were considered. Moreover, by examining the levels of LD on individual chromosomes, we were able to identify regions of the genome with unusually high LD. We found one chromosome – Chromosome 9 – that exhibited high LD, possibly due to the recent action of positive selection at loci on this chromosome. It is worth noting that high LD was found on Chromosome 9 in both Groups I and II, suggesting positive selection or some other causative factor has acted across a large geographic area that spans multiple subpopulations of this species.

Part of the reason we could detect unusual signatures of LD is that extensive recombination has occurred in *C. reinhardtii* during its evolutionary history. Historical recombination has led to the randomization of linked sites and a decrease in the baseline level of LD throughout the genome. Indeed, we estimate that more than 1,000 generations of outcrossing have occurred during the evolutionary history of the examined isolates. Our results definitively show that *C. reinhardtii* undergoes sex in nature. This finding has ramifications for general understanding of the ecology of this species, and suggests that genome-wide association mapping could be a useful technique in *C. reinhardtii* if a larger number of isolates are collected.

### Future studies of natural variation in *C. reinhardtii*

Natural genetic variation may provide new opportunities for using *C. reinhardtii* to study core features of eukaryotes, such as photosynthesis and the function and regulation of flagella, that cannot be examined in the other unicellular, eukaryotic model systems. However, multiple factors, including the availability of only small number of wild isolates and lack of a very closely related outgroup, presently limit population genomics and quantitative genetics research in this organism. Thus, our report represents only a starting point in the genome-wide characterization of *C. reinhardtii*'s diversity. Indeed, a more thorough assessment of the population structure and sequence variation of *C. reinhardtii* is needed. This will require the collection of a larger panel of isolates, which will ideally be obtained from across a broader geographic range and with more intensive local sampling. We have already begun to generate such a resource, and are optimistic that it will provide deeper insights into the extent and genetic mechanisms of ongoing adaptation and functional diversity in this important model organism.

## Methods

### Next generation sequencing of the isolates

DNA was extracted directly from samples of the isolates that were sent by the Chlamydomonas Resource Center (http://www.chlamycollection.org/) using Qiagen Plant DNeasy kits. We constructed sequencing libraries using the Nextera library construction kit (Illumina/Epicentre Biotechnology). We conducted the sequencing using a mixture of 50 base ×50 base paired-end reads from the University of Southern California Epigenome Center and 93 base ×93 base paired-end reads from the Beijing Genomics Institute. Sequencing data was deposited in the NCBI Short Read Archive under Project Accession PRJNA169374. Reads were mapped to the Joint Genome Institute v4 assembly of the *C. reinhardtii* genome. The sampe function of BWA was used to align the reads to the genome, and variants were identified using SAMTOOLS and BCFTOOLS [47], [48]. Each of the programs was run with its default settings. Only biallelic variants with a phred-like quality score greater than 30 were used in analyses, although we note that the use of other thresholds produced results that were qualitatively the same as those described in the paper. For our analyses, we focused on SNPs and invariant sites that had been directly sequenced in each of the strains. The most current annotation of the *C. reinhardtii* genome (Version 169) was obtained from Phytozome v4.3 (http://www.phytozome.net/) and used to assign sequenced sites to functional classes.

### Population structure analyses

The neighbor-joining analysis and PCA were conducted in R (http://www.r-project.org/). To construct the neighbor-joining tree, we used the ape and apTreeshape packages; the princomp function was used to conduct PCA. We also used STRUCTURE v2.3.2 to examine the strains for population subdivision [30]. We ran STRUCTURE across a range of *K* values from 2 to 4 with a burn in of 25,000 Markov Chain Monte Carlo (MCMC) cycles and 25,000 subsequent MCMC cycles. 25,000 SNPs were randomly chosen for use in the STRUCTURE analysis. We ran STRUCTURE three times for each *K* value, with the runs producing nearly identical results.

### Nonparametric correlations

Values of Spearman's ρ were computed using the cor.test function in R with method equal to ‘spearman.’ Latitudes of sampling were obtained from the Wikipedia pages for the cities in which strains were reportedly sampled (http://en.wikipedia.org).

### Nucleotide diversity analysis

Standard formulae for computing θ_W_ and θ_π_ were used [32], [33], [34], [35]. To obtain per base estimates of nucleotide diversity, we divided estimates of θ_W_ and θ_π_ by the total number of bases at which every isolate was sequenced. Similarly, site class-specific values were determined based on the number of sites of a particular class that had been sequenced.

### LD, recombination, and outcrossing analysis

A standard formula for computing the *r* ^2^ metric for LD was used [39]. The local recombination rate per generation (*r*) was estimated using the *C. reinhardtii* marker data and genetic map reported in [13]. Because the provided marker coordinates were based on an earlier version of the genome, we determined the positions of the markers relative to the v4 genome using the Basic Local Alignment Search Tool (BLAST) [49] in Phytozome. Specifically, primers that amplified markers that could be typed by PCR assays were BLASTed against the genome, and the best hit of each primer pair was recorded. We then estimated the genetic map positions of each genomic window by using chromosome-specific sigmoidal functions that were fit to the relationship between the physical map and genetic map positions of the markers in R. We estimated the population recombination rate (ρ) using the program LDHat v2.2 (http://ldhat.sourceforge.net; [42]). We analyzed each chromosome individually, and then estimated the global value of ρ as the average of the estimates for each chromosome. To determine the number of generations of outcrossing, we treated the N_e_ parameter in ρ as the effective number of generations of outcrossing that have contributed to the sample [41], and solved the equation N_e_ =  ρ/2*r*, where *r* was set at 1×10^−7^ Morgans per base.

## Supporting Information

Figure S1 **Principal component 3 is correlated with latitude of sampling.** Strains are colored by their subpopulation.
(PNG)

Figure S2 **Levels of nucleotide diversity across the genome measured independently for the two subpopulations.** Values for Group I strains are shown with darker colors, while values for Group II strains are shown with lighter colors.
(PNG)

Figure S3 **Linkage disequilibrium on Chromosome 9.** Chromosome-wide *r* ^2^ values are plotted for Chromosome 9 in the full isolate panel, Group I, and Group II in **A**, **B**, and **C**, respectively. A legend depicting the color scheme for the *r* ^2^ values is in the lower right quadrant. Data is plotted according to the physical order of SNPs on the chromosome (shown by the line y = x), with locations of SNPs on the chromosome indicated by small lines that connect the LD plots to the y = x line. The length of the chromosome is indicated in the plots. Red lines are used to show regions that have consistently high haplotype structure in the subpopulations.
(PNG)

Figure S4 **Local recombination rate throughout the genome, as estimated from a cross.** Local recombination rates were estimated based on data from a cross of two *C. reinhardtii* strains [13]. Each chromosome is color-coded. Note that estimates were not made for Chromosome 14 due to limited marker data for this chromosome. The regions of high LD on Chromosome 9 are shown with black bars.
(PNG)

Table S1 **SNPs detected on each chromosome by functional category.**
(XLS)
