# Supplementary figures and images for: Genome-Wide Characterization of Genetic Variation in the Unicellular, Green Alga Chlamydomonas reinhardtii
Source: PLoS One. 2012 Jul 25;7(7):e41307. doi: 10.1371/journal.pone.0041307 (PMC3405113; doi:10.1371/journal.pone.0041307)

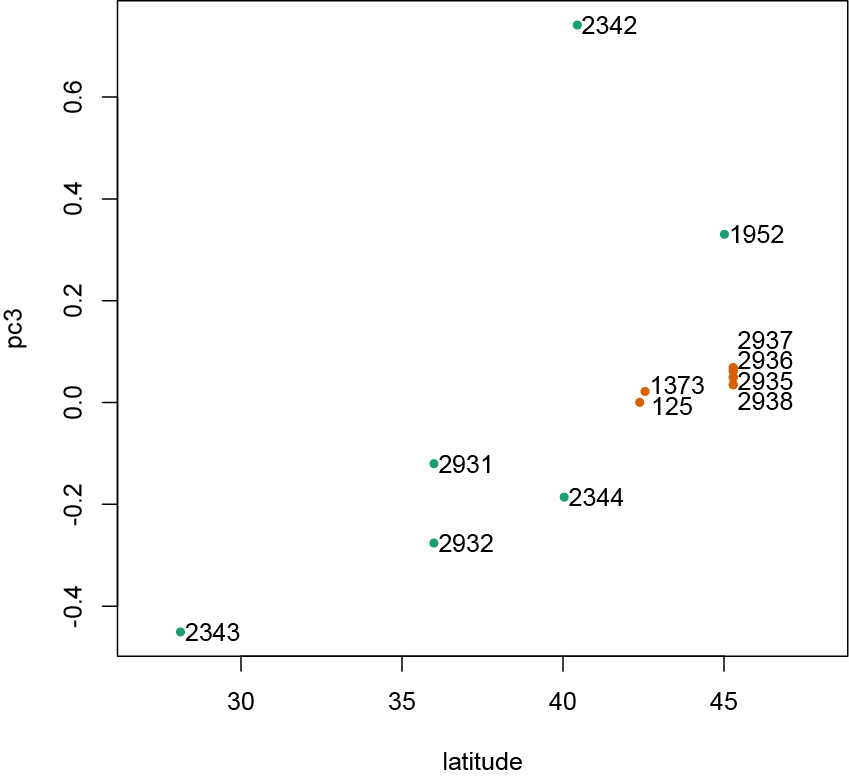

Supplement: Figure S1 — Principal component 3 is correlated with latitude of sampling. Strains are colored by their subpopulation. (PNG) [file pone.0041307.s001.png]

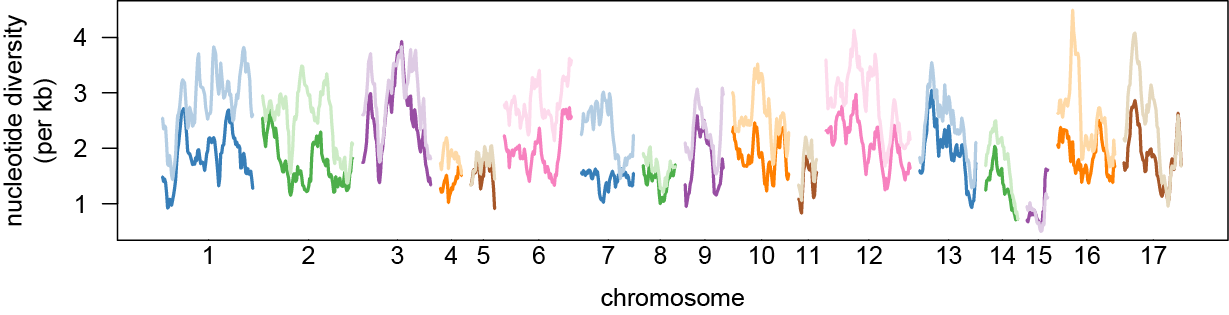

Supplement: Figure S2 — Levels of nucleotide diversity across the genome measured independently for the two subpopulations. Values for Group I strains are shown with darker colors, while values for Group II strains are shown with lighter colors. (PNG) [file pone.0041307.s002.png]

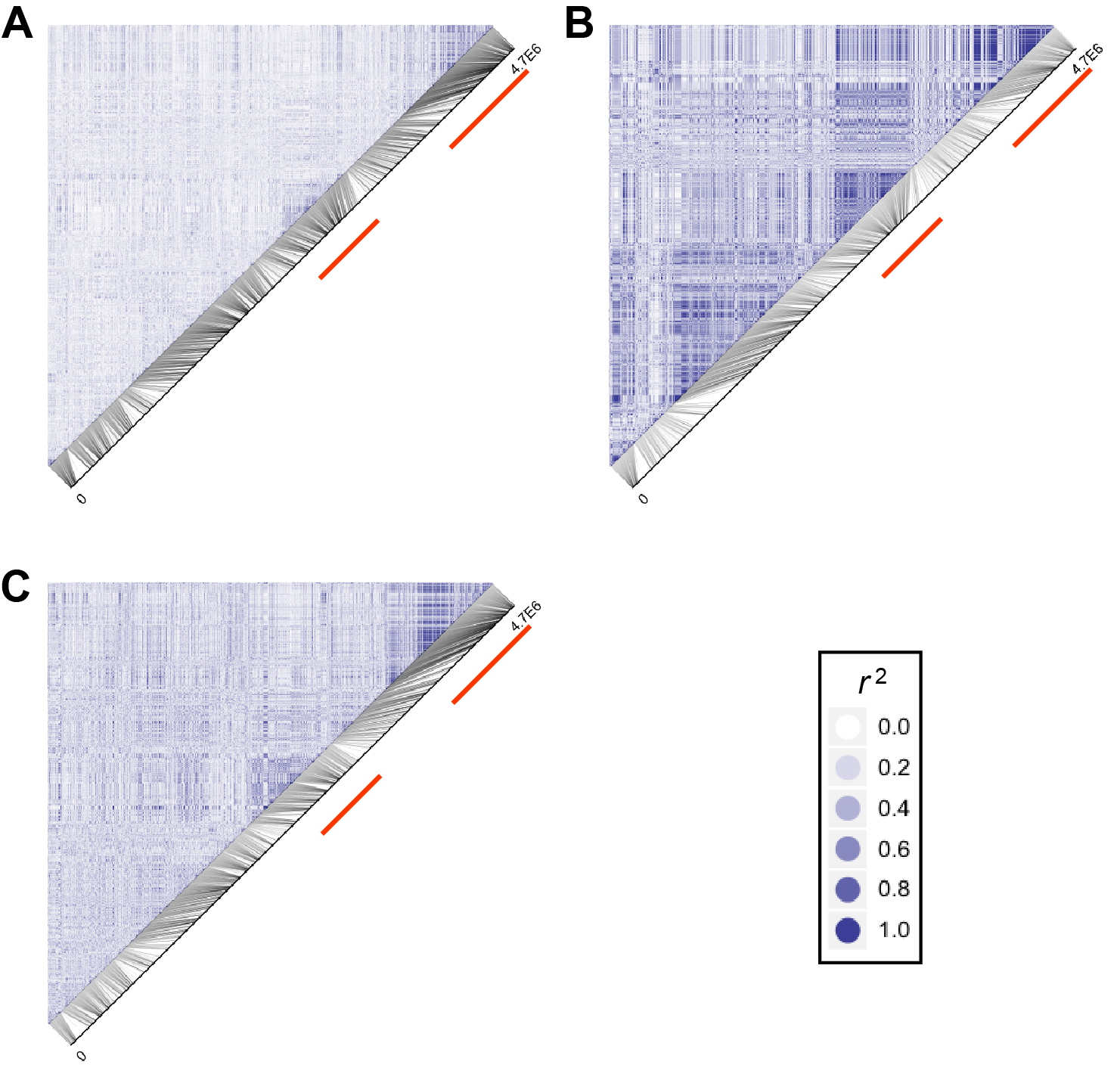

Supplement: Figure S3 — Linkage disequilibrium on Chromosome 9. Chromosome-wide r 2 values are plotted for Chromosome 9 in the full isolate panel, Group I, and Group II in A, B, and C, respectively. A legend depicting the color scheme for the r 2 values is in the lower right quadrant. Data is plotted according to the physical order of SNPs on the chromosome (shown by the line y = x), with locations of SNPs on the chromosome indicated by small lines that connect the LD plots to the y = x line. The length of the chromosome is indicated in the plots. Red lines are used to show regions that have consistently high haplotype structure in the subpopulations. (PNG) [file pone.0041307.s003.png]

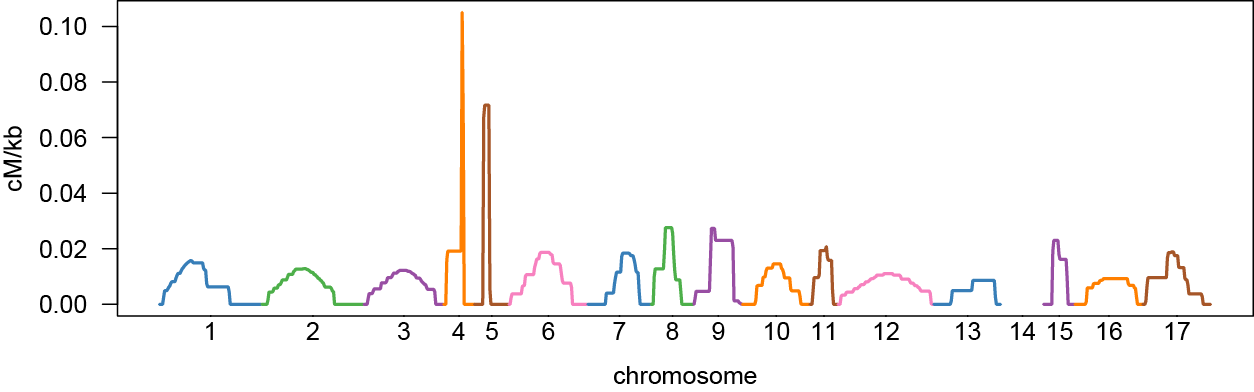

Supplement: Figure S4 — Local recombination rate throughout the genome, as estimated from a cross. Local recombination rates were estimated based on data from a cross of two C. reinhardtii strains [13]. Each chromosome is color-coded. Note that estimates were not made for Chromosome 14 due to limited marker data for this chromosome. The regions of high LD on Chromosome 9 are shown with black bars. (PNG) [file pone.0041307.s004.png]
